# Polycyclic aromatic hydrocarbons (PAHs), arsenic, chromium and lead in warty crab (*Eriphia verrucosa*): occurrence and risk assessment

**DOI:** 10.1007/s11356-021-14824-3

**Published:** 2021-06-15

**Authors:** Sara Lambiase, Andrea Ariano, Francesco Paolo Serpe, Marcello Scivicco, Salvatore Velotto, Mauro Esposito, Lorella Severino

**Affiliations:** 1 Istituto Zooprofilattico Sperimentale del Mezzogiorno, 80055 Portici, Italy; 2 Department of Veterinary Medicine and Animal Production, Division of Toxicology, University of Naples Federico II, 80137 Naples, Italy

**Keywords:** Heavy metal, PAH, *Eriphia verrucosa*, Estimated weekly intake, Incremental lifetime cancer risk

## Abstract

This study assesses the PAH and heavy metal levels in muscle of warty crabs (*Eriphia verrucosa*), from the northern coast of the Campania region improving the data on toxic contaminants in this crustacean. The results showed a minimal PAH contamination; the mean concentrations were as follows: 0.2, 1.6 and 1.7 μg kg^-1^ wet weight (ww) for BaP, PAH4 and PAH6, respectively. Regarding the levels of the two PAHs not included in the European regulations, the BkF mean concentration was 0.1 μg kg^-1^ ww, while DahA was detected only in 10.7% of samples. Pb and Cr were also detected at low levels with mean values of 0.068 and 0.468 mg kg^-1^ ww, respectively; instead, high As levels, with a mean value of 5.021 mg kg^-1^ ww, were found. Considering the EWIs and the ILCRs calculated in this study, the PAH, Pb and Cr contamination levels found in the edible part of the crabs resulted safe for human consumption. Contrariwise, the ILCR calculated for the As exceeded the acceptable level of cancer risk, although the calculation did not refer to the inorganic form which is the only one recognized as carcinogenic. Hence, this study shows that warty crabs can accumulate environmental contaminants in their muscle tissue representing an important route of exposure to these toxics for the local population that regularly consumes them. This finding highlights the importance of monitoring the presence of these pollutants in crabs and in general in all fish and seafood in order to ensure food safety for consumers.

## Introduction

Over the last decades, interest and awareness of institutional bodies, researchers and consumers in seafood safety have increased significantly. Fishes, mussels and crustaceans are part of the culinary traditions of several countries worldwide and represent an essential source of nutrients being rich in proteins, fatty acids, essential amino acids and vitamins (Cederholm 2017). Despite this, seafood can represent also a route of human exposure to dangerous chemical substances. Seafood safety is strictly linked to marine environment quality because many pollutants present in the aquatic environment can be bioaccumulated and biomagnified by marine organisms; therefore, concerns have been raised about the potential risks for human health derived by the consumption of contaminated fisheries products (Cappello et al. 2018). The Mediterranean Sea, as a semi-enclosed basin characterized by an intense naval traffic and industrial coastal activity, represents a geographic area highly sensitive to environmental pollution (Ferrante et al. 2018). Therefore, seafood from Mediterranean basin deserves to be carefully analysed to guarantee the safety of consumers and to provide reliable scientific data that can be exploited by the institutions to implement the panel of necessary analyses to maintain high standards of food safety and quality. Moreover, the monitoring of some aquatic species, because of their natural habitat, diet and position in the food chain, represents a useful bioindicator to collect data on the current health status of the marine ecosystem. *Eriphia verrucosa* is a benthonic species of crustacean, also called the warty crab, that lives in shallow waters up to the rocky coastlines. It is a common species in the Mediterranean Sea, regularly found along the Italian Tyrrhenian coasts, feeding primarily on bivalves, gastropods and polychaetes. Moreover, the warty crab is part of the traditional cuisine of southern Italy, especially of Campania region, and is widely consumed by the local population (Ariano et al. 2015). *Eriphia verrucosa* fishing takes place throughout the year and without limitations for both professional and not professional fishing. There are no minimum sizes to be respected for their fishing. The warty crab, because of its geographic distribution, position in the food web and consumption by humans, represents an optimal marine species for quali-quantitative toxicological investigations. Among numerous contaminants present in the marine environment, polycyclic aromatic hydrocarbons (PAHs) are persistent pollutants widely diffused, in particular in harbours, estuaries and coastal waters. They originate from incomplete combustion and pyrolysis of organic material, in processes as fossil fuel combustion, waste incineration and accidental oil spills (Tornero and Hanke 2016; Habibullah-Al-Mamun et al. 2019). PAHs are chemicals characterized by strong lipophilicity, solubility in organic solvents and high boiling and melting points. Living organisms can be exposed to PAHs through different routes, as inhalation or dermal contact, but primarily through ingestion that is considered the mainly way of exposure causing detrimental effects on animals and human health (Ferrante et al. 2018; Zaccaroni et al. 2018).

Based on the evidence of their toxic potential, European institutions have issued two regulations regarding presence of PAHs in food for human consumption: the Commission Regulation (EC) 1881/2006 2006and its amendment (Commission Regulation (EU) 835/2011 2011) that establishes the maximum levels (MLs) in molluscs and some smoked fish products of four PAH compounds (benzo[a]pyrene (BaP), benzo[a]anthracene (BaA), benzo[b]fluoranthene (BbF) and chrysene (Cry)) and the Commission Regulation (EC) 333/2007 2007and its amendment (Commission Regulation (EU) 835/2011 2011) that defines the sampling and analytical methods approved for PAH detection in food products. The need to officially assess the presence of PAHs in food items and to set MLs that safeguard the public health is linked to the high toxicity of these chemicals. The International Agency for Research on Cancer (IARC) listed sixteen different PAHs as dangerous compounds for human health due to their ability to be potentially carcinogens and mutagens (IARC (International Agency for Research on Cancer) 2013). Despite this, the EC regulation considers just four PAH compounds for which research in products intended for human consumption is mandatory. Moreover, EC regulation limits the research of PAHs only to two categories of fishery products: bivalve molluscs and muscle meat of smoked fish and smoked fishery products.

Among dangerous pollutants which can induce detrimental effects on human health, interfering with immune and reproductive systems, also trace elements can represent a risk for usual consumer of warty crabs. Although data regarding trace elements concentrations in warty crab are poor (Durmus et al. 2018; Zotti et al. 2016), Ariano et al. (2015) reported high concentrations of cadmium (Cd) in this crustacean (whole animal) that could lead to health risks for the population that usually consume this seafood. For this finding and considering that marine environment is affected by other toxic metals, this study also intends to assess the contamination levels of arsenic (As), chromium (Cr) and lead (Pb) which are elements very relevant for food safety. These metals are widespread and persistent pollutants which can be found at high concentrations in marine environment close to greatly urbanized and industrialized areas such as coastal areas, estuaries and river mouths (Maulvault et al. 2015). These elements are well known for their ability to induce harmful effects both in acute and chronic exposures; moreover, it has been reported that toxic metals as Pb can provoke severe health disease even at sub-lethal concentrations (Durmus et al. 2018). Human exposure occurs mainly through food consumption; in particular, fish and seafood are recognised as the mainly contributors to human As intake (Molin et al. 2015).

The aim of the present study is to evaluate PAHs, As, Cr and Pb concentrations in muscle of the warty crabs. In addition to the assessment of the four PAH compounds included in the European regulations for food safety and quality, we investigated also the presence of benzo[k]fluoranthene (BkF) and dibenzo[a,h]anthracene (DahA) which show a similar toxicity (Lundstedt et al. 2007; Spink et al. 2008).

## Materials and methods

### Biological material

**Fig. 1:**
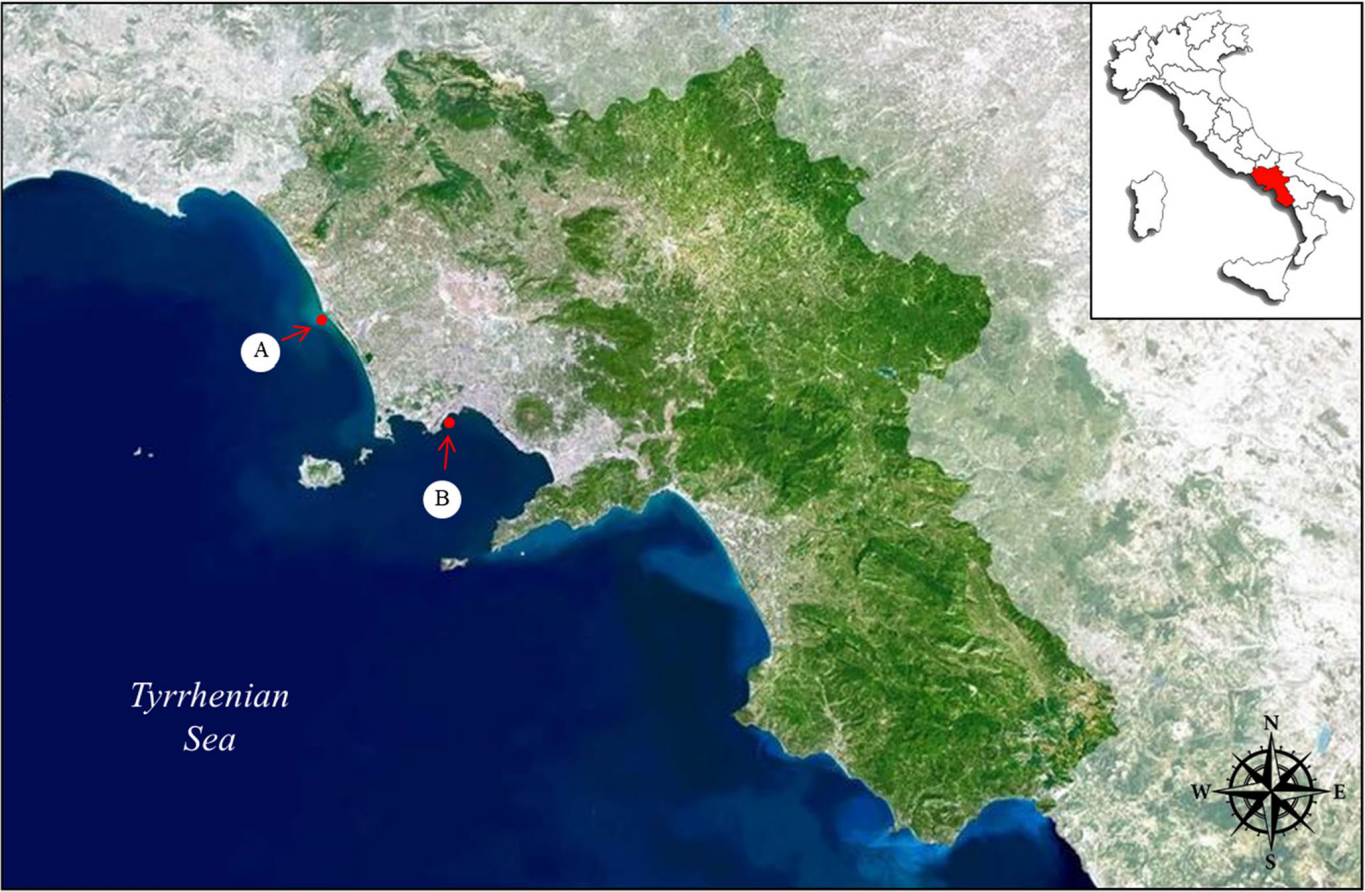
Map showing locations of the sampling sites: Castelvolturno (site A) and Naples (site B) in Campania region, Italy

Twenty-eight samples of warty crab (*Eriphia verrucosa*) were caught from two different locations, Castelvolturno (site A) and Naples (site B), located along the northern coast of the Campania region (Italy) (Fig. 1). All samples were collected between May and July 2016.

**Table 1:** PAH concentrations (range and mean ± SEM) in *E. verrucosa* expressed in μg kg^-1^ ww

	SITE A (n = 13)	SITE B (n = 15)
BaA	< LOQ–0.4	< LOQ–0.5
	(0.0 ± 0.03)	(0.1 ± 0.05)
Cry	< LOQ–1.8	< LOQ–1.2
	(0.7 ± 0.15)	(0.2 ± 0.11)
BbF	< LOQ–3.4	< LOQ–2.8
	(1.2 ± 0.27)	(0.6 ± 0.24)
BkF	< LOQ–0.4	< LOQ–0.4
	(0.1 ± 0.04)	(0.1 ± 0.03)
BaP	< LOQ–0.5	< LOQ–0.5
	(0.3 ± 0.07)	(0.1 ± 0.05)
DahA	< LOQ	< LOQ–0.4
	-	(0.1 ± 0.04)
PAH4	0.9–4.9	< LOQ–4.1
	(2.3 ± 0.33)	(1.0 ± 0.40)
PAH6	0.9–4.9	< LOQ–4.5
	(2.4 ± 0.36)	(1.1 ± 0.45)

Once captured, the crabs were euthanized at - 80 °C for 30 min. Then, the animals were weighed and measured using an Absolute Digimatic Caliper (Mitutoyo, Japan). The length (cl) and width (cw) of their carapace ranged between 3.8 and 6.0 cm (mean value: 4.9 cm) and 5.0 and 8.0 cm (mean value: 6.2 cm), respectively. Then the crabs were immediately sealed in decontaminated polyethylene bags, frozen at -20 °C and kept at the same temperature until delivery to the laboratory where they were dissected using steel tools including forceps, scissors straight and scalpels and analysed.

### Analysis of polycyclic aromatic hydrocarbons

Analysis of polycyclic aromatic hydrocarbons was performed according to the procedure described by Serpe et al. (2010). In brief, the crab muscle from claws and appendages was individually separated, homogenized and weighed (2.0 ± 0.5 g). Each sample was saponified with 10 mL of a solution of potassium hydroxide (2 N in ethanol) and liquid/liquid extracted for three times with 20 mL of cyclohexane. The extract was filtered, reduced to small volume and purified using a silica Sep-Pak cartridge and eluted with acetonitrile (ACN). The instrumental analysis were carried out by a high performance liquid chromatograph (HPLC) equipped with a fluorescence detector (Waters Alliance). Chromatographic separation was performed by an EnviroSep PP (125 × 3.2 mm, particle size 5 um, Phenomenex) LC column using the gradient elution with acetonitrile and ultrapure water as solvents at 0.5 mL min^-1^. The fluorescence detection was performed at the excitation and emission wavelengths of 294 and 404 nm, respectively. External standard method was used to determine PAH concentration in the samples. Linearity of method was checked by triple injection of standard solution at concentrations between 0.4 and 20.0 ng mL^-1^ obtaining a correlation coefficient (r^2^) at least 0.999. The calibration curve was made for every sequence of analysis. The limit of quantification (LOQ) was 0.2 μg kg^-1^ for each PAH.

### Analysis of heavy metals

Glassware and laboratory equipment were decontaminated before use with diluted ultrapure 65% HNO_3_ (ROMIL-UpA, Cambridge, UK) and were rinsed with Milli-Q water (Millipore Corp., Bedford, MA).

For the analysis, the crab samples (0.50 ± 0.02 g) were weighed in Teflon vessels with 5.0 mL of 69% HNO_3_ and 2.0 mL of 30% H_2_O_2_ (ROMIL-UpA) and placed in a microwave digestion system (Milestone, Bergamo, Italy). Microwave assisted digestion was performed with a mineralization program for 15 min at 190 °C. Then, the vessels were cooled at room temperature, and the digestion mixtures were diluted at the final volume of 50.0 mL by adding ultrapure water (resistivity 18.2 MΩ cm) that was produced in-house using a purification system arium® pro (Sartorius, Germany) (Ariano et al. 2019). Pb, Cr and As concentrations in the digested samples were determined with an atomic absorption spectrometer equipped with a graphite furnace and a L’vov platform (GF-AAS, Analyst 600, Perkin-Elmer, Bonenseewerk, Germany). The LOQs were 0.020, 0.050 and 0.165 mg kg^-1^ for Pb, Cr and As, respectively.

### Quality assurance

In the laboratory, appropriate quality assurance procedures were implemented in order to ensure the reliability of the results in accordance with the UNI/EN/ISO/IEC 17025 Standard (2005). Quality assurance and quality control (QA/QC) of the methods were monitored through analysis of procedural blanks, duplicate samples and standard solutions. Standard solutions of analytes were prepared from certified stock solutions containing Pb, Cr, As (atomic spectroscopy standard, Perkin Elmer) and the PAH of interest. Concentrations for each set of samples were determined in the medium range of the calibration curve. The performance of the method was assessed through participation in interlaboratory studies organized by FAPAS (Food Analysis Performance Assessment Scheme, Sand Hutton, UK).

### Statistical analysis

PAH concentrations were expressed in μg kg^-1^ wet weight (ww) as sum of BaA, Cry, BaP and BbF (PAH4) and sum of BaA, Cry, BaP, BbF, BkF and DahA (PAH6) using mean ± SEM (standard error of the mean). All metal concentrations were expressed in mg kg^-1^ ww as mean ± SEM.

Statistical significance of the influence of sampling sites (Castelvolturno Vs Napoli) has been tested using factorial analysis of variance. Furthermore, we apply ANOVA test to highlight differences between metals and PAH accumulation in the muscle of warty crabs and between the sampling areas. Multiple regressions have been used to discover statistical significance between metals and PAHs concentration and intrinsic variables (as length and width of specimens). One-sample Kolmogorov-Smirnov test confirmed normal distribution of our data. All our statistical analyses have been performed using MedCalc for Windows, version 18.11.3 (MedCalc Software, Ostend, Belgium). Significant value has been established at p <0.05.

For statistical calculations, the contribution of the undetected PAHs was considered equal to zero; for the undetected metals, it was considered a contribution equal to 0.5 LOQ (Menichini et al. 2004).

### Estimation of dietary intake and the carcinogenic risk

In order to assess the exposition to PAHs, Cr, As and Pb of the population that regularly consumes warty crabs coming from the coasts of the Campania region and thus to evaluate the potential health risk resulting from it, the estimation of the weekly intakes were calculated using the levels of contaminants determined in the crab muscles. The calculations were carried out only for adults as they are considered the main consumers of these crustaceans. The estimated weekly intakes (EWIs) are calculated using the equation described by Lambiase et al. (2017) and reported below:

$$ EWI=\frac{\left(C\times WI\right)}{BW} $$

where C is the mean concentrations of PAHs, Cr, As and Pb determined in crab samples; WI is the human weekly intake of crabs; and BW is the body weight (70 kg). The EWIs were calculated using both the WI of 37.7 g week^-1^ obtained by the Food and Agriculture Organization (FAO) (2013) and an estimated WI of one 100-g crustacean edible portion (Di Lena et al. 2018).

In addition, to assess the carcinogenic risk associated with the intake of PAHs, Cr, As and Pb through the consumption of local crabs, the incremental lifetime cancer risk (ILCR) is also calculated using the equation described by Tiwari et al. (2017):

$$ ILCR=\frac{ED\times EF\times EDI\times SF\times CF}{AT} $$

where ED is the exposure duration (83 year, Italian average life expectancy, (OECD/European Observatory on Health Systems and Policies 2017); EF is the exposure frequency (365 day yr^-1^); EDI is the estimated daily intake (ng kg^-1^ body weight (b.w.) day^-1^); SF is the oral cancer slope factor in kg day mg^-1^: 7.3 for BaP (Tiwari et al. 2017), 5 x 10^-1^ for Cr (Aendo et al. 2019), 1.50 for As (Li et al. 2011) and 8.5 x 10^-3^ for Pb (Aendo et al. 2019); CF is the conversion factor (1.0 × 10^−6^ mg ng^−1^); and AT is the average lifespan (30,295 days). The ILCR for PAH4 was calculated using the PAH concentration expressed in BaP equivalent obtained employing the toxic equivalent factors (TEFs) (Tiwari et al. 2017).

## Results

### Polycyclic aromatic hydrocarbons

**Fig. 2:**
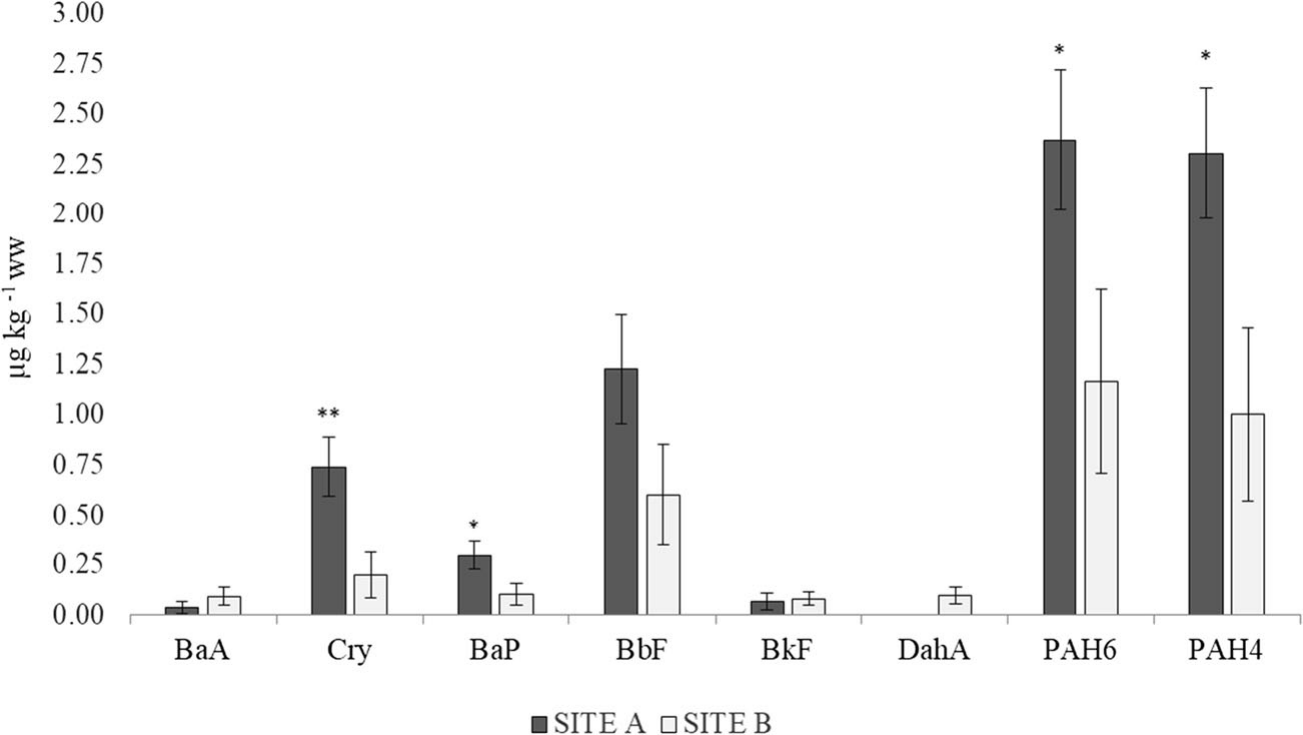
PAH concentrations in *E. verrucosa* depending on sampling sites: **A** Castelvolturno (n = 13) vs **B** Naples (n = 15). Vertical bars represent average concentration (μg kg^-1^ ww) ± SEM. Probability levels for significant differences: p<0.01 (**); p<0.05 (*)

PAH levels in crab muscle samples are reported in Table 1. The range concentrations of PAH4 and PAH6 were between < LOQ and 4.9 μg kg^-1^ ww for both the sums. Overall, the PAHs were detected in all samples except for seven crab muscles (25.0% of the total) that showed BaA, Cry, BaP and BbF concentrations below the LOQs. BaP was detected only in 39.3% of the total warty crabs, and its concentration ranged between < LOQ and 0.5 μg kg^-1^ ww; BkF and DahA were detected in 25.0% and 10.7% of the total samples, respectively, and their range concentrations were < LOQ and 0.4 μg kg^-1^ ww for both substances. The most abundant PAHs were BbF and Cry that contributed to PAH4 with 35.1% (mean: 0.9 μg kg^-1^ ww) and 21.6% (mean: 0.4 μg kg^-1^ ww), respectively. The levels of PAHs assessed in muscle of *E. verrucosa* varied between sampling sites (Fig. 2). Cry concentration was significantly higher in the crabs from Castelvolturno than those from Naples (p < 0.01); significant differences between site A and site B were also detected for BaP, PAH4 and PAH6 concentrations (p < 0.05).

**Table 2:** Metal concentrations (range and mean ± SEM) in *E. verrucosa* expressed in mg kg^-1^ ww

	SITE A (n = 13)	SITE B (n = 15)
Pb	< LOQ–0.240	< LOQ–0.242
	(0.056 ± 0.018)	(0.078 ± 0.016)
As	1.093–10.243	0.985–14.555
	(3.098 ± 0.850)	(6.688 ± 1.491)
Cr	< LOQ–3.216	< LOQ–2.318
	(0.712 ± 0.338)	(0.257 ± 0.150)

The analysed individuals varied in length and width. The multiple regression analyses indicate that there was no correlation between size and concentration of all analysed PAHs (p > 0.05).

### Heavy metals

**Fig. 3:**
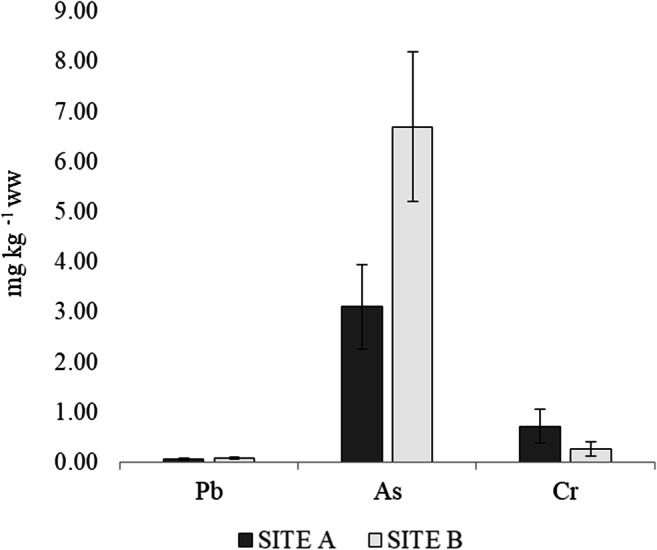
Heavy metal concentrations in *E. verrucosa* depending on sampling sites: **A** Castelvolturno (n = 13) vs **B** Naples (n = 15). Vertical bars represent average concentration (mg kg^-1^ ww) ± SEM. Probability levels for significant differences: p<0.01 (**); p<0.05 (*)

Regarding the occurrence of metals in crab muscles, the results are reported in Table 2. Arsenic was the most abundant element detected in all samples; its concentration ranged from 0.985 to 14.555 mg kg^-1^ ww. As was followed by Cr (range: < LOQ–3.216 mg kg^-1^ ww) and Pb (range: < LOQ–0.242 mg kg^-1^ ww). Contrary to PAHs, there were no statistical differences between metals concentration and sampling sites (p > 0.05) (Fig. 3). The multiple regression analyses indicate that there was no correlation between size and concentration of all analysed metals (p > 0.05).

## Discussion

### Polycyclic aromatic hydrocarbons

The crab muscle samples analysed in the current study showed low concentrations of each PAH investigated. The Commission Regulation (EU) 835/2011 2011 does not fix BaP and PAH4 MLs for crabs and in general for all crustaceans; therefore, it was not possible to assess whether the contamination levels determined in the samples were compliant with the EU Regulation. Nevertheless, the BaP and PAH4 concentrations reported herein resulted lower than the MLs set for smoked crabs and for other types of foodstuffs (Commission Regulation (EU) 835/2011 2011).

In addition to lacking of European and national regulations as regard PAH contamination in these marine organisms, there are also very few data available on this issue in literature which are also often reported in different way (dry or wet weight) making the comparisons difficult. Nevertheless, in order to assess the PAH contamination level of the crabs coming from the northern coast of Campania region in relation to crustaceans coming from other marine areas and hence the potential health risk of the consumption of this seafood, the results reported in this study were compared to the data described by few authors on crabs coming from other coastal areas. Abdolahpur Monikh et al. (2014) described BaP concentrations in muscles of *Portunus pelagicus,* sampled in the Persian Gulf that ranged from 170 to 956 ng g^-1^ on dry weight (dw) (mean: 200 ng g^-1^ dw). Considering that crustaceans have an average water content of about 80% (Vinogradov 2018), the mean BaP concentration expressed on wet weight became 40 ng g^-1^ ww, and therefore it was much higher than the mean values of BaP ​​found in the present study. Zhang et al. (2020), in a study on PAH bioaccumulation in marine organisms from South Yellow Sea in China, determined in crab samples concentrations (sum of 16 PAHs) that ranged from 119.11 to 223.34 ng g^-1^ dw resulting higher than our results.

Moreover, they described that among benthic and benthivorous organisms, crabs showed lower PAH concentrations than shrimps and demersal fish. The mean PAH concentrations found in this study resulted instead higher than those found in crabs (*Callinectes amnicola*) from Atlas Cove (Nigeria) analysed by Olayinka et al. (2019) who determined concentrations of the PAH6 below the detection limits in all samples. Perugini et al. (2007), in Norway lobster from Central Adriatic Sea, also showed Cry and BbF concentrations below the detection limit, while the BaA and BkF were slightly higher than the concentrations detected in this study. Therefore, the overall PAH level reported herein resulted comparable or lower than those described in other marine areas indicating a low risk for human health. In order to obtain more data on the bioaccumulation of PAHs in crustaceans from the coasts of the Campania region and the health risks for the population that consumes this food, the concentrations found in this study were compared with those reported by other authors that investigated this marine area. From comparing, the PAH levels detected in warty crabs resulted lower than the levels found in other fish and seafood species (Fasano et al. 2018; Fiorito et al. 2019).

The low levels of PAHs detected in the present study suggest that the exposure to these contaminants of the population of this area that consumes crabs and in general crustaceans and the consequent health risk are also low. All these findings led to suppose that the PAH concentrations found in the edible parts of the crabs can be considered at baseline levels.

It was interesting to find that the PAH concentrations determined in the crab muscles from Castelvolturno were significantly higher than those found in the animals from Naples; this difference in concentrations could be attributed to the presence of the Volturno river that flows into the Tyrrhenian Sea at Castelvolturno.

The Volturno is the longest Southern Italian river which crosses densely populated areas, such as the province of Caserta, and that collects pollutants mainly from zoo-technical and agricultural activities, handcrafts and industries (Isidori et al. 2004; Zuzolo et al., 2016).

Otherwise, the source of contamination of the Gulf of Naples is mainly represented by maritime transports, fishing and coastal tourism (Esposito et al. 2017). Regarding the difference in PAH concentrations in relation to the size of crabs, it was not statistically significant in agreement to the results showed by other authors (Perugini et al. 2007).

As a concern, BkF and DahA were detected in crab muscles at levels comparable to PAHs included in Commission Regulation (EU) No 835/2011. BkF and DahA are classified by the IARC as possible and probable carcinogenic to humans, respectively.

On the basis of this classification, the EFSA Panel on Contaminants in the Food Chain (CONTAM Panel) included also these two substances in the group of eight PAHs that are considered the only indicators of the carcinogenic potency of these contaminants in food (EFSA (European Food Safety Authority) 2008). Therefore, considering the BkF and DahA levels described herein in crabs, it may be recommended to develop new food safety plans to monitor also these two substances.

### Heavy metals

The heavy metal analysis carried out in this study showed low levels of Pb and Cr but a high presence of As in crab muscles samples. Interestingly and contrary to what was observed for PAHs, the heavy metal levels found in the crabs were higher than those found in fish and mussels coming from the same marine area (Fasano et al. 2018) showing that these organisms, as also described by other authors, can bioaccumulate toxic elements in their tissues when they live in polluted environment (Perry et al. 2015; Karar et al. 2019; Bordon et al. 2020). Pb was detected at low levels in the crabs from both sampling sites resulting below MLs established in muscle meat of crustaceans (0.5 μg g^-1^ ww) by the Commission Regulation (EC) No 1881/2006 and its amendment (Commission Regulation (EU) No 420/2011, 2011). Concerning Cr and As, although some forms of these elements are recognized as carcinogenic to humans (Group 1) by the IARC (IARC, 1973, 1980, 2012), there are no MLs laid down for food by the European Commission.

Overall, As was the most abundant element detected in warty crab muscles followed by Cr and Pb. Comparison of the studies carried out by other authors showed that the As concentrations assessed in warty crabs from northern coast of Campania region resulted higher than those measured in the muscle of *Eriphia verrucosa* and *Rapana venosa* from Turkey (Levent and Öztekin 2016), in the muscle of fiddler crab of *Uca tangeri* species (mean: 1.76 μg g^-1^) collected from Spain (Suner et al. 1999) and also in the edible muscle of warty crab from the Black Sea that had As concentrations ranging from 1.34 μg g^-1^ to 2.43 μg g^-1^ ww (Durmus et al. 2018). It has been reported that benthonic species that feed close to the coasts may bioaccumulate higher As levels than pelagic ones. In fact, being As naturally presents in rocks, marine environment near the coasts has an abundant amount of this element (Ramos-Miras 2019). In particular, Campania region is a territory characterised by high background levels of As of volcanic origin; in fact, high concentrations of this metal were found in the pyroclastic deposits in the NW and SE sectors of the region, including the coastal areas, as also in the Volturno River plain (Albanese et al. 2007; Petrik et al. 2018). On the basis of this information, it was possible to hypothesize that the As levels found in the crabs analysed herein derived from natural sources due to the large volcanic area present in the region.

Moreover, it is important considered that of the total As (tAs) amount only the inorganic As (iAs) rate is harmful to human health. According to the data available in literature and the EFSA opinion, in fish and in general all seafood, the tAs include mainly arsenobetaine, and the iAs rate varies depending on the species of fish or seafood (EFSA, 2014). However, Cubadda et al. (2016), in a study on the dietary exposure of the Italian population to iAs, found that crustaceans and molluscs are one of the food group with the highest iAs concentration (28.3 ng g^-1^ ww).

Concerning the other two heavy metals measured, the Pb concentrations found in the muscle of *E. verrucosa* in Naples and Castelvolturno sites were approximatively comparable than those measured in the muscle of warty crab from Turkey (Levent and Öztekin 2016) and from Adriatic Sea (Zotti et al. 2016). Instead, the levels of Pb resulted lower than the concentrations detected in muscles of *Rapana venosa* (0.1 to 0.7 μg g^-1^) analysed by Mülayim and Balkıs (2015) and in the edible muscle of warty crab (0.13 μg g^-1^ to 0.36 μg g^-1^ ww) analysed by Durmus et al. (2018) both collected from the Black Sea. The Pb levels assessed in this study resulted also lower than the levels found in the edible muscle (0.10 μg g^-1^) of Chinese mitten crabs (*Eriocheir sinensis*) from rivers and lakes of Netherlands (Hoogenboom et al. 2015), in muscles of the blue crab (1.08 ± 0.56 mg kg^-1^) collected from the northern Bay of Bengal (Karar et al. 2019) and in muscles of the red crab from the Gulf of Mexico (Perry et al. 2015).

For Cr, the levels detected in the present study were comparable to those found in muscle of *Rapana venosa* (0.47 ± 0.01 μg g^-1^) from the Black Sea (Topcuoğlu et al. 2002) and in muscles of the blue crabs (0.68 ± 0.50 mg kg^-1^) collected from the northern Bay of Bengal (Karar et al. 2019), while the Cr levels resulted higher than those found in muscle of *Rapana venosa* (0.1 to 0.2 μg g^-1^) from the Black Sea analysed by Mülayim and Balkıs (2015) and in muscle of warty crab from Adriatic Sea (Zotti et al. 2016). Moreover, it has been reported by many authors that the bioaccumulation of toxic metals in crabs, and in general in all marine animals, depends on several physiological and biometric factors among which the body size is recognized as an important parameter (Pinheiro et al. 2012; Knutsen et al. 2018; Wiech et al. 2020). In the current study, the statistical analysis showed that the heavy metal levels were not statistically correlated to the size of the crabs (p > 0.05), suggesting that these parameters have a minor effect on metals accumulation in subjects inside the size range considered in this study. In fact, the length and width of warty crabs carapace in the present study ranged between 3.8 and 6.0 cm (mean value: 4.9 cm) and 5.0 and 8.0 cm (mean value: 6.2 cm), respectively. It has been described that the metal bioaccumulation is strongly influenced by metabolism in fish (Canli and Atli 2003). Hence, same authors have been suggested that the negative correlation is probably due to a faster metabolism rate of the smaller animals, which correspond to the younger specimens, than the older ones (Sofoulaki et al. 2018). Therefore, these processes could lead to a dilution of the contaminant concentration with growth. However, it has been observed that the negative correlation between metal concentrations and body size occurs when the marine pollution is at low levels; for high levels of pollution, instead, a positive correlation has been described (Sofoulaki et al. 2018).

### Health risk assessment

The EWI calculated for BaP, PAH4, Cr, As and Pb that occurs through the consumption of crabs from the coasts of the Campania region are showed in Table 3. As regard PAHs, a human tolerable weekly intake has not been fixed. The EFSA Panel on Contaminants in the Food Chain (CONTAM) in its Scientific Opinion regarding polycyclic aromatic hydrocarbons in food (EFSA (European Food Safety Authority) 2008) reported median values of consumer exposure to BaP and PAH4 for the food category fish and fishery products of 21 and 170 ng day^-1^, respectively. The EWIs calculated using the values reported by EFSA (European Food Safety Authority) 2008assuming a body weight of 70 kg (2.1 ng kg^-1^ b.w. per week for BaP and 17 ng kg^-1^ b.w. per week for PAH4) were higher than the EWIs calculated with the concentrations found in the present study. This finding showed that the exposure of the population to these contaminants through the consumption of local crabs involves a low health risk. Moreover, for human exposure risk characterization, in its opinion, EFSA used the bench mark dose lower confidence limit (BMDL_10_) for a 10% increase in the number of tumour in animals (EFSA (European Food Safety Authority) 2008). The BMDL_10_ derived by EFSA (European Food Safety Authority) (2008) were 0.07 and 0.34 mg kg^-1^ b.w. day^-1^ for BaP and PAH4, respectively. Also considering these values, the consumption of local crabs resulting safe for human health. Regarding Cr, As and Pb, the CONTAM Panel established a TDI only for Cr (III), supposing that all chromium in food is in this chemical form, that is 0.3 mg kg^-1^ b.w. per day (2.1 mg kg^-1^ b.w. per week), resulting higher than the value calculated herein. For As and Pb, the EFSA Panel has not been set any TDI or TWI values. Precisely, the Joint FAO/WHO Expert Committee on Food Additives (JECFA) fixed a provisional tolerable weekly intake (PTWI) of 15 μg kg^-1^ b.w. per week for iAs and 25 μg kg^-1^ b.w. per week for Pb. These values were considered no longer suitable by the EFSA Panel that established for iAs a BMDL_01_ between 0.3 and 8 μg kg^-1^ b.w. per day for an increased risk of cancer of the lung, skin and bladder, as well as skin lesions (EFSA, 2014) and for Pb a BMDL_01_ of 1.50 μg kg^-1^ b.w. per day for an increased risk of cardiovascular effects and nephrotoxicity in adults (EFSA, 2012). Hence, as for the PAHs, the EWI values calculated for the metals showed a low exposure to these toxic pollutants for the population that consumes crustaceans.

**Table 3:** Estimated weekly intake (EWI) of BaP, PAH4, Cr, As and Pb calculated using both WIs (37.7 and 100 g week^-1^) and expressed in μg kg^-1^ b.w. week^-1^

	BaP	PAH4	Cr	As	Pb
EWI (WI 37.7 g week^-1^)^a^					
Min	nc	nc	0.0135	0.5305	0.0054
Max	0.0003	0.0026	1.7320	7.8389	0.1303
Mean	0.0001	0.0009	0.2523	2.7041	0.0366
Median	nc	0.0007	0.0439	0.9193	0.0304
EWI (WI 100 g week^-1^)^b^					
Min	nc	nc	0.0357	1.4071	0.0143
Max	0.0008	0.0070	4.5943	20.7929	0.3457
Mean	0.0003	0.0023	0.6691	7.1728	0.0970
Median	nc	0.0019	0.1164	2.4386	0.0807

^a^WI obtained from FAO, 2013

^b^WI obtained from Di Lena et al. 2018

*nc* Not calculable

Regarding the carcinogenic risk associated with the BaP, PAH4, Cr, As and Pb intakes through the consumption of crabs, the ILCRs calculated are reported in Table 4. For the carcinogenic risk assessment, it was set a threshold of 1.0 x 10^-6^ which means there is one in a million chances for an individual to develop cancer over a lifetime as a result of exposure to a carcinogen (Tiwari et al. 2017; Aendo et al. 2019); at this level, the cancer risk is considered negligible. The risk becomes serious when the ILCR exceeds the threshold of 1.0 x 10^-4^ (Aendo et al. 2019).

**Table 4:** Incremental lifetime cancer risk (ILCR) of BaP, PAH4, Cr, As and Pb calculated using both daily intakes (DIs, 5.38 and 14.28 g day^-1^)

	BaP	PAH4^c^	Cr	As	Pb
ILCR (DI 5.38 g day^-1^)^a^					
Min	nc	nc	9.61 x 10^-7^	1.14 x 10^-4^	6.53 x 10^-9^
Max	3.03 x 10^-7^	4.91 x 10^-7^	1.24 x 10^-4^	1.68 x 10^-3^	1.58 x 10^-7^
Mean	1.06 x 10^-7^	1.62 x 10^-7^	1.80 x 10^-5^	5.79 x 10^-4^	4.44 x 10^-8^
Median	nc	5.88 x 10^-8^	3.13 x 10^-6^	1.97 x 10^-4^	3.69 x 10^-8^
ILRC (DI 14.28 g day^-1^)^b^					
Min	nc	nc	2.55 x 10^-6^	3.02 x 10^-4^	1.74 x 10^-8^
Max	8.05 x 10^-7^	1.30 x 10^-6^	3.28 x 10^-4^	4.46 x 10^-3^	4.20 x 10^-7^
Mean	2.82 x 10^-7^	4.30 x 10^-7^	4.78 x 10^-5^	1.54 x 10^-3^	1.18 x 10^-7^
Median	nc	1.56 x 10^-7^	8.32 x 10^-6^	5.23 x 10^-4^	9.81 x 10^-8^

^a^DI obtained from FAO, 2013

^b^DI obtained from Di Lena et al. 2018

^c^The PAH concentrations used for the ILCR calculations were expressed in BaP equivalent

*nc* Not calculable

The ILCRs calculated in this study were in the range between 4.46 x 10^-3^ and 6.53 x 10^-9^. The highest ILCR values, which exceed the threshold of 1.0 x 10^-4^, were obtained for As and for Cr, when the ILCR was calculated using the maximum Cr concentration found in the crabs, indicating that for these elements, there is a potentially risk for human health. However, these results were obtained using the total concentration of As and Cr and not the rates recognised as carcinogenic, which are iAs and Cr (VI); hence, due to the lack of information, it was impossible to make toxicological discussions.

## Conclusions

This study assesses the PAH and heavy metal levels in *E. verrucosa* from the northern coast of the Campania region improving the data regarding dangerous chemical compounds in this traditional Mediterranean crustacean. The results suggested a limited contamination of Pb, Cr and PAHs in the study areas indicating a low risk for human consumption. In fact, the EWIs and ILCRs calculated for these carcinogens were compliant with the thresholds considered safe for human health. Moreover, in addition to the four PAHs included in the Commission Regulation (EU) No 835/2011, the analysis showed the occurrence also of BkF and DahA in crab muscles. Considering that these two substances are recognized as possible and probable carcinogenic to humans respectively, it would be necessary to put more careful attention to official controls and monitoring on toxicological investigation including also these two PAHs to assure public health. Contrariwise, higher As concentrations that had probably a natural origin were found in muscle of warty crabs. The ILCRs calculated for As exceeded the acceptable level of cancer risk indicating a potentially threat for human health. These findings should be thoroughly studied in order to understand the bioaccumulation mechanisms and to identify anthropogenic sources of As pollution in the these marine areas in addition to natural ones.

## Data Availability

The datasets used and/or analysed during the current study are available from the corresponding author on reasonable request.
